# Prevalence and correlates of intimate partner violence among family planning clients in Conakry, Guinea

**DOI:** 10.1186/s13104-015-1811-7

**Published:** 2015-12-23

**Authors:** Alexandre Delamou, Ghazaleh Samandari, Bienvenu Salim Camara, Pernamou Traore, Fatoumata Guilinty Diallo, Sita Millimono, Defa Wane, Maimouna Toliver, Kira Laffe, Fabio Verani

**Affiliations:** Centre National de formation et de recherche en santé rurale de Maferinyah, Forécariah, Guinea; Department of Public Health, Faculty of Medicine, University of Conakry, Conakry, Guinea; EngenderHealth, New York, USA; Association Guinéenne pour le Bien-Etre Familial, Conakry, Guinea; Ministry of Health, Conakry, Guinea; EngenderHealth, Conakry, Guinea

**Keywords:** Intimate partner violence, Family planning, Prevalence, Correlates, Factors, Patterns, Guinea

## Abstract

**Background:**

Intimate partner violence (IPV) is a global public health problem that affects women’s physical, mental, sexual and reproductive health. Very little data on IPV experience and FP use is available in resource-poor settings, such as in West Africa. The aim of this study was to describe the prevalence, patterns and correlates of IPV among clients of an adult Family Planning clinic in Conakry, Guinea.

**Methods:**

The study data was collected for four months (March to June 2014) from women’s family planning charts and from an IPV screening form at the Adult Family Planning and Reproductive Health Clinic of “Association Guinéenne pour le Bien-Etre Familial”, a non-profit organization in Conakry, Guinea. 232 women out of 245 women who attended the clinic for services during the study period were screened for IPV and were included in this study.

**Results:**

Of the 232 women screened, 213 (92 %) experienced IPV in one form or another at some point in their lifetime. 169 women reported psychological violence (79.3 %), 145 reported sexual violence (68.1 %) and 103 reported physical violence (48.4 %). Nearly a quarter of women reported joint occurrence of the three forms of violence(24 %).Half of the IPV positive women were current users of family planning (51.2 %) and of these, 77.9 % preferred injectable contraceptives. The odds of experiencing IPV was higher in women with secondary or vocational level of education than those with higher level of education (AOR: 8.4; 95 % CI 1.2–58.5). Women residing in other communes of Conakry (AOR: 5.6; 95 % CI 1.4–22.9) and those preferring injectable FP methods (AOR: 4.5; 95 % CI 1.2–16.8) were more likely to experience lifetime IPV.

**Conclusions:**

IPV is prevalent among family planning clients in Conakry, Guinea where nine out of ten women screened in the AGBEF adult clinic reported having experienced one or another type of IPV. A holistic approach that includes promotion of women’s rights and gender equality, existence of laws and policies is needed to prevent and respond to IPV, effective implementation of policies and laws, and access to quality IPV services in Guinea and countries with higher rates of IPV.

## Background

Intimate partner violence (IPV) is a serious violation of one’s human rights and a public health problem that affects women’s physical, mental, sexual and reproductive health worldwide [1, 2]. According to a recent World Health Organization (WHO)’s report, more than one in three women (35.6 %) globally and 36.6 % in Africa have experienced physical and/or sexual violence [3, 4]. The lifetime physical and/or sexual IPV prevalence has been reported to vary from 15 to 71 % in ten countries worldwide [3]. Intimate partners perpetrate more than a third of female homicides, worldwide [5]. Survivors of IPV are less likely to use reproductive health services [6] and more likely to experience adverse reproductive health outcomes such as low birth weight infants, pre-term delivery and neonatal death [1].

The prevalence of IPV among women has already been documented in different settings using population based studies [7–10], women living with HIV [11, 12] and those whom are pregnant [13, 14]. Furthermore, a few studies including data from West African countries suggest that women experience a high rate of physical or sexual violence, up to 76 % of women [15, 16]. Additionally, previous studies have examined the use of contraceptives among women who experienced IPV [17, 18]. However, very little empirical data exist on the experience of IPV in women in West Africa, generally, and Guinea, specifically. Furthermore, these data are typically gathered in the context of violent political conflict and do not capture the contraceptive pattern of survivors. Guinea has a low contraceptive prevalence rate (7 %) that has not improved since 1999, and the maternal mortality ratio is estimated to be 724 per 100,000 Live Births [19, 20], indicating a need to improve family planning services overall, and in particular for women struggling with IPV. By examining IPV experience among a sample of family planning clients, we hope to better understand characteristics of women experiencing violence in this population and their preferred patterns of contraception.

From January to June 2014, a pilot project for the integration of IPV screening and counseling into FP services was implemented in the adult family planning clinic of the Association Guineenne pour le Bien-etre Familial (AGBEF), a member association of the International Planned Parenthood Association (IPPF) in Conakry, Guinea [21].

In this study, we report on: (1) the sociodemographic characteristics of IPV among family planning clients, (2) the types of IPV experienced by family planning clients and, (3) factors associated with types of IPV among clients.

## Methods

### Study design

The data for this study were derived from a pilot project conducted by the RESPOND Project, led by EngenderHealth and in partnership with AGBEF. This project piloted the integration of IPV screening and counseling into existing family planning services [21].

The study data was collected for four months (March to June 2014) from women’s family planning charts and from an IPV screening form at the Adult Family Planning and Reproductive Health Clinic of AGBEF, a non-profit organization in Conakry, Guinea.

### Study setting

#### Study site

AGBEF is a Member Association of the International Planned Parenthood Federation (IPPF). The organization operates 9 clinics in Guinea, including one youth clinic and one adult clinic in Conakry. The pilot IPV-FP integration approach was implemented at the adult family planning clinic in Conakry. The clinic staff included a nurse and midwife in charge of FP provision, antenatal care and sexually transmitted infections (STI) screening and care; a counselor who provided counseling and family planning methods to clients and support/administrative staff.

#### The Guinea IPV pilot project

The project aimed at supporting improved reproductive health (RH) by integrating IPV screening and counseling into FP services. The integration approach was based on creating a safe and enabling environment in which clients felt comfortable disclosing their experience(s) of IPV. It built on the GATHER (Greet, Ask, Tell, Help, Explain and plan the Return) Model for Family Planning Counseling [22] which the AGBEF clinic was using and staff was familiar with, providing additional consideration for IPV screening and services referral. This approach combined the basic tenets of respectful and informed client interaction with guidance on where and how to integrate IPV screening and counseling.

The main interventions of the project included conducting formative research; developing an IPV-FP integration curriculum; field testing the curriculum; providing the clinic with follow-up technical assistance and, evaluating the results of the pilot project. Full documentation of the RESPOND IPV pilot project is described elsewhere [21].

### Study population

All women of reproductive age attending the clinic and seeking family planning services (new and current users) were eligible for recruitment. Women providing informed consent were screened for IPV. In total, 245 women attended the clinic for services during that study period. Of these, 232 consented to be screened for IPV and were included in data collection.

### Data variables, sources of data and data collection

The IPV screening questionnaire was composed of nine questions informed by different screening question instruments [23, 24]. IPV was defined in this study as any experience of abusive behaviors and actions perpetrated by any intimate partner during their lifetime, and included psychological, physical and sexual abuse. Sociodemographic characteristics included age (in years), marital status (married/cohabitated or not married -single, divorced, or widow), occupation (housewife, workwoman, seller, employee or student), occupation of woman’s partner (unemployed, workwoman, seller, employee or student), woman and her partner’s level of education (none, primary, secondary/vocational or university), residence (Dixinn commune or other communes), number of live children. Other variables included contraceptive method use (injectable, pills, Intra-uterine device, IUD or implants), reasons for choosing the method (discretion, fertility return, not constraining or easy to use) and duration of FP use. For data collection, first an IPV documentation form was used after the counseling session by the midwife/nurse to screen the client for IPV and to classify the behavior as psychological, physical or sexual, a combination of two, or all three forms of IPV. Second, the women’s medical records were used to extract socio-demographic information and family planning use.

### Data analysis

Data collected from family planning clients was double entered, by two independent encoders, into a file using EpiData Entry software (version 3.1, EpiData Association, Odense, Denmark). The two data files were compared and discordances resolved by cross-checking with the source documents. Data was analysed using STATA 13 software (STATA Corporation, College Station, TX, USA). Frequencies (%) were calculated to describe women’s characteristics, contraceptive use and IPV types. Pearson’s Chi square or Student *t* test were used, respectively, to compare proportions or means of variables with a level of significance set at P = 0.05 and a 95 % confidence intervals. Sociodemographic factors and contraceptive use correlated with lifetime prevalence of any type of IPV were assessed using logistic regression. All study variables were considered “a priori” for inclusion in the logistic regression model and contraceptive use was dichotomized (injectable versus non injectable). The adequacy of the final model was tested by the Hosmer and Lemeshow test for goodness of fit. Unadjusted and adjusted odds ratios and their 95 % CI were derived with a significance level set at 5 %.

### Ethics approval

The study protocol was approved by the National Ethics Committee for Health Research of Guinea. Written informed consent was obtained from all participants prior to IPV screening. All interviews were conducted in complete privacy and data collection tools were strictly anonymous. A safety plan was discussed and elaborated with women found IPV positive and referral to a partner local organization offered care and follow up for victims of gender-based violence was discussed with women [21].

## Results

Overall, 245 women attending the AGBEF adult FP/RH clinic in Conakry were provided counseling and IPV screening during the study period. Of them, 232 women provided an informed consent and accepted screening and 213 out of them were screened positive for IPV, meaning they have experienced one or more form of IPV.

### Sociodemographic characteristics

The sociodemographic characteristics of the women are presented in Table 1. The mean age of participants was 28.5 years (±7.9 SD), 60 % were unmarried, and the majority of women had none or primary level of education (55.4 %). The mean number of pregnancies were 2.8 (±2.0 SD) and the mean number of live children was 2.4 (±1.8 SD). Women reported that their intimate partners were mainly business owners (33.3 %) or employed by others (28.2 %), and most had secondary to higher level of education (54.5 %).

**Table 1:** Socio-demographics characteristics of family planning clients screened for Intimate partner violence (IPV) at the AGBEF Clinic from March to June 2014 in Conakry, Guinea

Variables	Screened for IPV number (%)	IPV positive number (%)
Total	232	213
Age, years		
15–19	27 (11.6)	24 (11.3)
20–24	61 (26.3)	58 (27.2)
25–34	81 (34.9)	74 (34.7)
35–49	63 (27.2)	57 (26.8)
Mean (SD)	28.7 (8.0)	28.5 (7.9)
Marital status		
Married	94 (40.5)	86 (40.4)
Not married	138 (59.5)	127 (59.6)
Occupation		
Housewife	21 (9.1)	20 (9.4)
Workwoman	40 (17.2)	38 (17.8)
Seller	85 (36.6)	80 (37.6)
Employee	36 (15.5)	32 (15.0)
Student	50 (21.6)	43 (20.2)
Level of education		
None	73 (31.5)	67 (31.5)
Primary	55 (23.7)	51 (23.9)
Secondary and vocational school	74 (31.9)	71 (33.3)
University	30 (12.9)	24 (11.3)
Residence		
Dixinn	105 (45.3)	102 (47.9)
Other communes	127 (54.7)	111 (52.1)
Partner occupation		
Unemployed	37 (15.9)	32 (15.0)
Workman	38 (16.4)	35 (16.4)
Seller	77 (33.2)	71 (33.3)
Employee	64 (27.6)	60 (28.2)
Student	16 (06.9)	15 (07.1)
Partner’s level of education		
None	68 (29.3)	64 (30.0)
Primary	38 (16.4)	33 (15.5)
Secondary and vocational school	77 (33.2)	70 (32.9)
University	49 (21.1)	46 (21.6)
Number of pregnancies		
0	28 (12.0)	23 (10.8)
1	51 (22.0)	48 (22.5)
2–4	109 (47.0)	101 (47.4)
≥5	44 (19.0)	41 (19.3)
Mean (SD)	2.7 (2.1)	2.8 (2.0)
Number of live children		
0	30 (12.9)	25 (11.7)
1	59 (25.4)	56 (26.3)
2–4	112 (48.3)	103 (48.4)
≥5	31 (13.4)	29 (13.6)
Mean (SD)	2.4 (1.8)	2.4 (1.8)
Previous use of FP		
No	115 (49.6)	104 (48.8)
Yes	117 (50.4)	109 (51.2)
Mean duration of FP use (SD), months	6.6 (10.3)	6.9 (10.6)
Previous FP method used (n = 130)		
Injectable	98 (75.4)	95 (77.9)
Pills	06 (04.6)	06 (04.9)
IUD	22 (16.9)	18 (14.7)
Implants	04 (03.1)	03 (02.5)
FP method used at this visit		
Injectable	174 (75.0)	166 (77.9)
Pills	10 (04.3)	09 (04.2)
IUD	39 (16.8)	31 (14.6)
Implants	09 (03.9)	07 (03.3)
Reasons for choosing this FP method		
Discretion	80 (71.4)	75 (71.4)
Fertility return	11 (09.8)	11 (10.5)
Not constraining	16 (14.3)	14 (13.3)
Easy	05 (04.5)	05 (04.8)

### Lifetime prevalence and types of violence

In this study, 213 (92 %, 95 % CI 88.5–95.5 %) of the respondents experienced IPV in one form or another at some point in their lifetime. 169 women reported psychological violence (79.3 %), 145 reported sexual violence (68.1 %) and 103 reported physical violence (48.4 %), (Table 2).

**Table 2:** Prevalence of Intimate partner violence (IPV) among women screened at the AGBEF Clinic from March to June 2014 in Conakry, Guinea

	Among women screened for IPV N (%)	Among IPV positive women N (%)
Total	232	213
Prevalence of IPV		
IPV negative	19 (8.2)	N/A
IPV positive	213 (91.8)	N/A
Type of IPV		
Psychological violence		169 (79.3)
Physical violence		103 (48.4)
Sexual violence		145 (68.1)

**Fig. 1:**
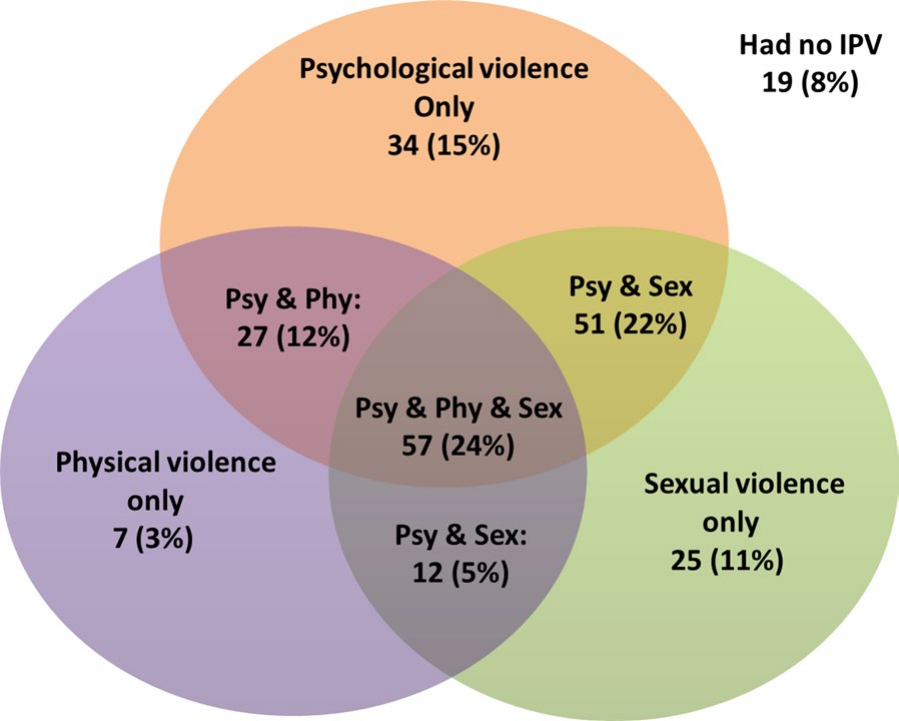
Venn diagram illustrating overlaps between lifetime experiences of psychological, physical and sexual violence reported by FP clients in Conakry, Guinea, March to June 2014

Figure 1 shows the patterns of the joint occurrences of different forms of IPV among family planning clients screened. Psychological violence (34 women, 15 %) and sexual violence (11 %) were more likely to occur in isolated form compared to physical violence (3 %). 147 women (63 %) experienced more than one type of IPV. The joint occurrence of the three forms of violence was the most common (57 women, 24 %) followed by the joint occurrence of psychological and sexual violence (51 women, 22 %).

### Family planning practices

Half of the 213 women who were IPV positive were current users of family planning (51.2 %) with a remarkable preference for injectable contraceptives (77.9 %). The majority of women preferred injectable methods irrespective of their IPV status (positive or negative) and of the type of violence experienced (psychological, physical and sexual). The main reasons for preferring injectable were discretion (75 women, 71 %), lack of constraints (14 women, 13 %) and fertility return (11 women, 10.5 %).

### Correlates of lifetime IPV

Table 3 shows the results of the unadjusted and adjusted logistic regression modelling performed to identify the correlates of lifetime experience of IPV.

**Table 3:** Correlates of any type of IPV among FP clients at the AGBEF Clinic from March to June 2014, Conakry, Guinea (N = 232)

	IPV positive N (%)	Unadjusted OR (95 % CI)	Adjusted^a^ OR (95 % CI)	P-value
Total	232	232	232	
Age, years	28.5 (7.9)	0.98 (0.92–1.03)	0.93 (0.82–1.05)	0.222
Marital status				0.954
Married	86 (91.5)	1	1	
Not married	127 (92.0)	1.07 (0.41–2.78)	0.96 (0.23–3.92)	
Woman’s level of education				
None	67 (91.8)	2.79 (0.82–9.49)	0.75 (0.06–9.02)	0.820
Primary	51 (92.7)	3.19 (0.82–12.36)	1.59 (0.18–14.31)	0.681
Secondary and vocational	71 (95.9)	*5.92 (1.37–25.51)*	*8.44 (1.22–58.47)*	0.031
Higher	24 (80.0)	1	1	
Woman’s occupation				
Housewife	20 (95.2)	3.26 (0.37–28.27)	12.92 (0.44–72.38)	0.139
Workwoman	38 (95.0)	3.09 (0.60–15.80)	7.18 (0.51–100.21)	0.143
Seller	80 (94.1)	2.60 (0.78–8.70)	9.69 (0.77–99.46)	0.078
Employee	32 (88.9)	1.30 (0.35–4.83)	2.56 (0.36–18.33)	0.349
Student	43 (86.0)	1	1	
Residence				0.016
Dixinn	111 (87.4)	1	1	
Other communes	102 (97.1)	*4.90 (1.39–17.31)*	*5.60 (1.37–22.85)*	
Partner occupation				
Unemployed	32 (86.5)	1	1	0.291
Workman	35 (92.1)	1.82 (0.40–8.25)	2.83 (0.41–19.50)	0.306
Seller	71 (92.2)	1.85 (0.52–6.51)	2.33 (0.46–11.85)	0.269
Employee	60 (93.8)	2.34 (0.59–9.34)	2.75 (0.46–16.64)	0.783
Student	15 (93.8)	2.34 (0.25–21.86)	0.63 (0.02–16.28)	
Partner’s level of education				
None	64 (94.1)	2.42 (0.61–9.64)	3.77 (0.57–24.68)	0.166
Primary	33 (86.8)	1	1	0.859
Secondary and vocational	70 (90.9)	1.51 (0.45–5.13)	0.87 (0.18–4.22)	0.105
Higher	46 (93.9)	2.32 (0.52–10.41)	7.00 (0.67–73.74)	
Number of live children	2.45 (1.8)	1.16 (0.87–1.55)	1.55 (0.85–2.85)	0.155
FP method adopted				
Injectable	166 (95.4)	*4.86 (1.85–12.77)*	*4.54 (1.22–16.83)*	0.024
Non injectable	47 (81.0)	1	1	
Duration of FP use (months)	6.9 (10.6)	1.04 (0.97–1.11)	1.08 (0.99–1.19)	0.093

Hosmer–Lemeshow test for goodness-of-fit: chi2 (8 d.f.) = 7.64; p = 0.470

Woman’s occupation and Partner’s occupation were not included in bivariate and multivariate analyses because of correlation with respectively woman and partner’s level of education

*OR* odds ratios, *CI* confidence interval, *IUD* intra uterine device

^a^Multivariate analysis adjusting for possible confounding factors

### Unadjusted analysis

In bivariate analyses, lifetime IPV was significantly associated with woman’s level of education, residence of the woman, and family planning method used. Women with a secondary to vocational level of education were more likely to experience lifetime IPV than women with higher level of education (Odds ratio (OR) 5.9; 95 % Confidence Interval (95 % CI) 1.4–25.5)

The odds of experiencing IPV was higher among women residing in other communes of Conakry (OR 4.9; 95 % CI 1.4–17.3) compared with the Dixinn Commune where the AGBEF clinic was established. Finally, women who preferred injectable as FP method were more likely to experience IPV compared with those who preferred non injectable FP methods such as implants, IUD or pills (OR 4.9; 95 % CI 1.9–12.8).

### Adjusted analysis

After adjusting for possible confounding factors (Table 3), lifetime IPV remained significantly associated with woman’s level of education, residence of the woman, and family planning method used.

The adjusted odds ratio (AOR) of experiencing IPV remained higher and even increased in women with secondary to vocational level of education as compared to those with higher level of education (AOR: 8.4; 95 % CI 1.2–58.5). Women residing in other communes of Conakry were more likely to experience lifetime IPV than women living in the Dixinn commune (AOR: 5.6; 95 % CI 1.4–22.9). The odds of lifetime IPV remained higher in women preferring injectable FP methods as compared to women using non injectable methods (AOR: 4.5; 95 % CI 1.2–16.8). Finally the results suggested an association between duration of FP use and lifetime occurrence of IPV (an 8 % increase for each additional month of use) but the association was not statistically significant (p = 0.093).

## Discussion

This is one of the first studies reporting on the prevalence and correlates of IPV among family planning clients in West Africa, and specifically in Guinea. Overall, the study showed high prevalence of IPV among the study population (92 %). The prevalence observed in this study is greater than that reported in previous studies conducted on IPV across Africa, including Guinea [3, 8, 25, 26]. While psychological violence was the most common IPV type experienced by women (79 %), our results show that 63 % of those screened experienced more than one type of IPV and almost one out of four women experienced the three types of IPV at some time in their life. Injectable contraceptive were the preferred family planning methods used by women, with discretion being the main reason for such choice.

After adjusting for possible confounding factors, lifetime IPV was significantly associated with woman having a secondary to vocational level of education, residence in communes other than Dixinn commune which hosts the FP clinic, and the use of injectable FP methods.

Our findings raise issues that merit further discussion.

First, the high prevalence observed in our study may be attributable to differences in measurement or it may be that women seeking FP services are particularly high risk, as seen in other studies [3, 8, 17, 18]. For instance our sample included only women seeking family planning services in one clinic setting. The lifetime prevalence of IPV in women aged 15 to 49 years was estimated to be 62 % in 2009 in Guinea [25]. The WHO population-based household surveys reported a lifetime prevalence of physical or sexual partner violence, or both, varying from 15 to 71 % [3]. In a study by Alio et al. [17], women who had experienced IPV were significantly more likely to report having used contraception compared with women who had not experienced IPV (OR 1.30, 95 % CI 1.22–1.38). In addition, contraceptive use has been reported to be 1.5 to 2 times higher in women exposed to IPV compared to women who were not in Jordan [18] and in Bangladesh [27].

Second, 78 % of women in our study preferred injectable because they thought it was a discreet method. Women using injectable FP methods were more likely to experience lifetime IPV compared to those using non injectable methods in our context. This suggests that women who seek injectable may be in violent relationships and therefore want to hide their contraceptive use from their partner to avoid future violence.

Previous reports have emphasized the fact that violence against women is still considered in many settings as normal including by women themselves [19, 28]. For instance the Guinean 2012 Demographic and Health Survey (DHS 2012) [29] reported that more than half of women in the country believe a man is justified in beating his wife if she argues with him (77.6 %), goes out without his permission (82.7 %), or refuses sexual intercourse (69.7 %). In the DHS 2012, only 28 % of women said a woman is justified in refusing sexual intercourse with her husband no matter what her reason is. In such a context, using a discreet and concealable method—such as injectable—that is fully controlled by the woman enables her to safeguard her sexual and reproductive health, and achieve her contraceptive goals. Contraceptive methods other than the injectable, such as pills and implants, are more visible, more difficult to conceal, or easily detected by the male partner. They were therefore less preferred by victims of IPV as their discovery could lead to the resumption of and/or an increase in violence [18, 28]. Despite being a discrete method, IUD use was not associated with higher risk of IPV probably because it is not widely used in the country as is injectable [29].

Our study has some limitations. The sample size was small and the study period was short, which limit generalization of the findings to all family planning clients in Guinea. Furthermore, this was a cross-sectional study that does not allow exploring causality or verifying the answers provided by respondents. Finally, we did not screen for recent (i.e. previous 12 months) experience of IPV, and instead captured violence at any point in their relationship, which could have inflated reports of IPV compared to other study instruments, especially considering that almost 70 % of women in our sample were aged 25 years and more. Despite these limitations, our findings provide evidence of the high prevalence of IPV among FP clients in Guinea.

## Conclusion

IPV is prevalent among family planning clients in Conakry, Guinea where nine out of ten women screened in the AGBEF adult clinic reported having experienced one or another type of IPV. This is among the highest findings reported to date. The overlapping of the three types of violence accounted for 24 % and was the most common reported feature of IPV. More efforts are needed to address this situation through a holistic approach that includes promotion of women’s rights and gender equality, sexual and gender based violence (SGBV) prevention programming, existence of laws and policies to prevent and respond to SGBV, effective implementation of policies and laws, and access to quality SGBV services (including legal, psychosocial, and health) in Guinea and countries with higher rates of IPV [30, 31].

Further work is needed to understand the process of IPV and evaluate the impact of interventions aimed at screening and or managing IPV in public and private health settings.

## Abbreviations

AGBEF: Association Guinéenne pour le Bien-Etre Familial (Guinean Family Wellbeing Association)
AOR: adjusted odds ratio
CI: confidence intervals
IPV: intimate partner violence
IPPF: International Planned Parenthood Federation
IUD: intra uterine device
OR: odds ratio
FP: family planning
SGBV: sexual and gender based violence
STI: sexually transmitted infections
WHO: World Health Organisation
